# Congenital Stridor

**Published:** 1900-12-08

**Authors:** 


### CONGENITAL STRIDOR.

Dr. John Thomson and Dr. A. Logan Turner[1] have recently made some interesting observations in regard to that very curious condition, the congenital stridor of infants. In a typical case they say the infant, who appears normal in other respects, is noticed shortly after birth to have noisy breathing. The noise consists of a croaking sound accompanying inspiration, which rises to a high-pitched crow when a longer or more vigorous breath is taken. Expiration may be accompanied by a short croak when the stridor is loud, but at other times it is noiseless. Even in the most severe cases there are occasional brief intervals during which there is no sound audible; but with this exception the stridor goes on constantly while the child is awake, and sometimes even when asleep. Anything that causes deeper breathing, such, for example, as exposure to colder air or the effort of sucking, is apt to intensify the sound. The child's power of crying and coughing is unaffected, and notwithstanding the apparent obstruction to the inspiration, and the marked inspiratory indrawing of the thoracic and abdominal walls, there is not the slightest sign of distress nor is there any cyanosis. There has been considerable difference of opinion as to the cause of the stridor in these cases, the site of the obstruction having been variously thought to be in the pharynx, at the upper aperture of the larynx, at the cords, and in the trachea at the level of the thymus.

After a careful examination, on the one hand of the peculiarities of respiration in very young children, and, on the other, of the conformation of the larynx in stridor cases, the authors arrive at the conclusion that the primary element in the causation of this condition is a disturbance of the co-ordination of the respiratory movements, probably due to some developmental backwardness of the cortical structures which control them; that such changes of form as are found in the larynx is merely an exaggeration of the normal infantile type, and is mainly, if not entirely, the result of a constantly recurring sucking-in of the upper aperture of the soft larynx, which is induced by the ill co-ordinated and spasmodic nature of the breathing; that there is no proof of the existence of any congenital malformation of the laryngeal aperture in these cases; and that such deformity is not necessary to account for the symptoms, seeing that even normal babies may crow in a very similar manner when they are coming out of chloroform; that the sounds are not produced in the pharynx, but probably originate partly in the larynx proper and partly at the abnormally approximated ary-epiglottic folds. Lastly, that the neurosis by which the symptoms are caused has not seemed in the cases observed to depend on the presence of adenoid growths or other causes of reflex irritation.

The essence, however, of the position taken up by the authors of this paper is that they regard the peculiar conformation of the upper aperture of the wind-pipe found in these cases not as a congenital malformation to which is to be attributed the whole train of symptoms, but as an acquired deformity which, as well as the stridor, is the result of the ill co-ordinated stammering breathing. The point is perhaps of no great practical importance, except that by placing this curious and sometimes very alarming condition in line with other "stammerings," it removes from the practitioner the temptation, which must sometimes be very strong within him, to do something radical for the relief of the little patient.

[1] Brit. Med. Jour., Dec. 1.

